# Can Embodied Contemplative Practices Accelerate Resilience Training and Trauma Recovery?

**DOI:** 10.3389/fnhum.2018.00134

**Published:** 2018-04-11

**Authors:** Joseph J. Loizzo

**Affiliations:** Nalanda Institute for Contemplative Science and Weill Cornell Medical College, New York, NY, United States

**Keywords:** resilience, trauma, mindfulness, imagery, yoga breathing, polyvagal theory, vagal tone, tantra

Since meditation research has increasingly focused on methods like mindfulness, we have come to see contemplative practice in general as taking a top-down approach to lowering stress and building resilience (Hölzel et al., 2011). Yet given the key role body awareness plays in mindfulness, and its use of interoception to reduce stress and boost resilience, one could make a case for re-conceiving it as a somatic practice along the lines of Hatha yoga (Loizzo, 2016b). Regardless of how we view mindfulness, there are several less familiar forms of contemplative practice beyond posture yoga and mindful breathing that take a largely somatic approach to health and well-being (Loizzo, 2016a). Much of my work in mind/body research and contemplative psychotherapy involves these lesser known, less studied forms I call “embodied contemplative practices” (Loizzo et al., 2017). My aim in this piece is to suggest that these embodied practices are ripe for basic and clinical research on somatic approaches to resilience, both because recent findings have begun to shed light on their long obscure paths of action, and because as a group they target the deeper neural structures and networks that most hinder or promote affective and somatic reliance.

Embodied contemplative practice consists of two broad types of methods: top-down and bottom-up. Both work synergistically on the subcortical networks supporting implicit memory and procedural conditioning to calm the post-traumatic reactivity and reflex stress-reactivity that most powerfully block resilience (Loizzo, 2014). Top-down embodied practices include corrective body-imagery (visualization) and prosocial prosody (recitation); bottom-up embodied practices include expressive movement (posture-gesture) and energizing breath-control (kundalini yoga). A small body of research on these methods gathered since the 1950's has yielded compelling findings which suggest that they may be the most effective of all contemplative practices (Das and Gastaut, 1955; Benson et al., 1990; Lazar et al., 2000; Amihai and Kozhevnikov, 2014). While we are far from having a critical mass of data and even further from any consensus on these techniques, several lines of research have converged recently that shed light on their key mechanisms and effects and raise the promise of this fertile field growing to maturity in the coming years.

The main lines of advancement that promise to unlock this field have come in research on mental imagery (Kosslyn et al., 2006), exceptional performance (Brown, 2009), embodied cognition (Cardona, 2017), and autonomic regulation (Porges, 2011). Among these, most crucial is the new understanding of the evolution and function of the autonomic nervous system (ANS) articulated by Stephen Porges' polyvagal theory. This theory focuses on the autonomic adaptations that evolved in the mammalian transition to support the growing capacity for social engagement that gave our vulnerable ancestors a selective advantage. In brief, the new, myelinated “smart” vagus that co-evolved with modified auditory, facial, and glossopharyngeal nerves and with mammalian neuropeptides oxytocin/vasopressin allows mammals to maintain a socially engaged mode of brainstem regulation that modulates primitive sympathetic and parasympathetic stress-reactivity and supports the full capacity of the new social brain. This nuanced view of the ANS further explains that the new suite of cranial nerves supports a range of abilities—voluntary breathing, fine control of vocalization and facial expressions, enhanced sensitivity to vocal tones—that work together to sustain the key facets of social communication, at the same time as they cross-activate the smart vagal nerve and ventral vagal complex that modulate primitive stress-reactivity.

This simple yet powerful theory for the first time helps clarify why the unusual array of top-down and bottom-up embodied contemplative methods have historically been combined into one integrated practice, traditionally called Tantric Yoga or simply Tantra (Loizzo, 2012, 2016a). Vividly imagining the caring face and body image of a mentor or archetype in visualization stimulates the mirror-neuron mediated empathy network and evokes prosocial memories and imprints of one's own caring face and body, cross-activating the facial and vagal nerves (Ekman et al., 1990; Porges, 2011). Imagining or expressing soothing vocal tones in prayer or chanting stimulates glossopharygeal and auditory feedbacks that cross-activate the ventral vagal complex (Lazar et al., 2000; Porges, 2011). Voluntary rhythmic breathing activates the smart vagus and its medullary nucleus, regulating reflex autonomic stress-reactivity (Brown and Gerbarg, 2005a; Shannahoff-Khalsa, 2008). And finally, gentle, rhythmic movement and deep abdominal breathing activate feedbacks sent to the solitary nucleus from visceral vagal afferents, promoting the bottom-up regulation of primal stress reactivity (Brown and Gerbarg, 2005a; Loizzo, 2016a).

The first point to highlight about this simple yet powerful theory is that it is key to understanding the biology of human resilience. The foundation of resilience for mammals is our newly evolved capacity to regulate primal stress reactivity by exercising the top-down regulative structures of the mammalian brain—including self-regulating regions of the prefrontal cortex, cingulate cortex, and hippocampus (Porges, 2011).

The second point to highlight about this theory is that it helps lay bare the autonomic underpinnings of the neurobiology of trauma and post-traumatic stress-reactivity, the greatest obstacle to mind/body resilience. Given that the optimal mode of neural processing for healthy social engagement—left hemispheric dominance with top-down prefrontal control—depends on consistently high levels of smart vagal activity, while the traumatic mode of neural processing—right hemispheric dissociation with bottom-up amygdalar/hypothalamic drive—depends on heightened sympathetic and/or primitive vagal tone, the neurobiology of traumatic stress-reactivity blocking resilience is intimately linked to failures in smart vagal modulation of the ANS (Davidson, 2000; Porges, 2011).

This brings us to the potential clinical application of embodied contemplative practice. Current research in trauma care—also recently come of age—has recognized that the stress-reactive dissociation of traumatic experience from prefrontal modulation and left-sided verbal processing makes trauma resistant to conventional psychotherapy (van der Kolk, 2003). As a result, leading trauma researchers and clinicians are moving toward less discursive, more somatic approaches, including psychodramatic re-enactments, narrative and poetic reframing, posture yoga and intensive breath-work (Van der Kolk, 2015). This recent paradigm shift clearly suggests an overlap between current advances in trauma care and the mechanisms and effects of embodied contemplative practice, raising the potential of an effective trauma intervention based on these techniques. Initial studies of posture yoga and intensive breath-work in veterans and trauma survivors have already been done, showing some promise (Brown and Gerbarg, 2005b; Seppälä et al., 2014).

If, as the science suggests, embodied contemplative methods are more targeted than mindfulness, self-compassion, or simple yoga at removing stress-reactive blocks and building resilience at the level of implicit memory, body memory, and procedural learning, there is reason to believe that these techniques may be more effective in treating trauma than other contemplative methods, or than the latest somatic approaches. The fact that embodied practices combine top-down with bottom-up methods, and integrate all four strategies to boost and maintain smart vagal tone suggests that they may well prove more effective at treating trauma and building resilience than approaches that rely on one method alone (Loizzo et al., 2009). In addition, since these practices involve positive dramatic imagery, heroic narrative, poetic recitation, breath-induced flow states, and empowering posture and gesture, all in a concerted, portable, and structured practice system, they may also prove more feasible and sustainable than current conventional or alternative trauma therapies (Kozhevnikov et al., 2009; Loizzo et al., 2017).

Finally, given the growing recognition that the neurobiology of trauma is not restricted to major life stressors or obvious traumas, but applies across the whole range of chronic stressors and microtraumas experienced in childhood and everyday life, the potential uses of embodied contemplative practice may extend to the larger domain of mental health and public health. Given their targeted action, one would expect embodied methods to extend the general stress-reducing, resilience-building impact which methods like mindfulness and compassion exert at the cortical level (Klimecki et al., 2012; Tang et al., 2015) to the subcortical level of implicit memory, body memory, and procedural conditioning. The potential impact of thus deepening the broad public health benefits of yoga, mindfulness, and compassion training can be gauged by considering the toll which destructive emotions and subclinical traumatic stress-reactivity have on our homes, schools, workplaces, and civil society. In my opinion, embodied contemplative practices are ripe for both groundbreaking research on somatic approaches to resilience and for powerful applications in the clinic and everyday life.

## Author contributions

The author confirms being the sole contributor of this work and approved it for publication.

### Conflict of interest statement

The author declares that the research was conducted in the absence of any commercial or financial relationships that could be construed as a potential conflict of interest.
